# A Case of Paraneoplastic Neurological Syndrome Leading to the Diagnosis of Large Cell Neuroendocrine Carcinoma From Opsoclonus-Myoclonus Syndrome

**DOI:** 10.7759/cureus.48911

**Published:** 2023-11-16

**Authors:** Takashi Saito, Akiko Maeda, Hiroaki Nagano, Tomoo Kishaba

**Affiliations:** 1 Department of Neurology, Japanese Red Cross Shizuoka Hospital, Shizuoka, JPN; 2 Department of General Internal Medicine, Okinawa Chubu Hospital, Uruma, JPN; 3 Department of Respiratory Medicine, Aso Iizuka Hospital, Iizuka, JPN; 4 Department of Respiratory Medicine, Okinawa Chubu Hospital, Uruma, JPN; 5 Department of Home and Lifestyle Medicine, Ikigai Home Clinic, Okinawa, JPN

**Keywords:** literature review, case report, refractory, opsoclonus-myoclonus syndrome, large-cell neuroendocrine carcinoma, paraneoplastic neurological syndrome

## Abstract

Opsoclonus-myoclonus syndrome (OMS) is a rare neurological disorder characterized by myoclonus, ataxia, and tremors. It can be classified as neoplastic or idiopathic, with small cell lung cancer being commonly associated. Herein, we present a rare case of refractory paraneoplastic neurological syndrome (PNS) caused by large cell neuroendocrine carcinoma (LCNEC), a rare form of non-small cell lung cancer (NSCLC). A 60-year-old otherwise healthy man presented with acute-onset dysarthria, gait instability, and numbness on the right side of his body. According to the clinical symptoms and neurological examination, we initially suspected cerebellar infarction; however, brain imaging revealed no abnormal findings. After a few days, the patient developed worsening horizontal nystagmus, irregular ocular rhythms, and generalized involuntary movements, indicative of OMS. A systemic evaluation revealed a solitary nodule in the lower lobe of the right lung, leading to a clinical diagnosis of PNS. The patient underwent segmentectomy to treat an early-stage LCNEC nodule after one month from onset. Despite all therapeutic interventions, OMS was refractory, and after consulting with the person himself and the family, palliative care was selected. However, the patient showed a clinical response belatedly five months after surgery. This case highlights the importance of considering PNS, and that it may be associated with a rare malignancy when cerebellar symptoms are observed, and the challenges in managing refractory PNS associated with rare forms of NSCLC.

## Introduction

Opsoclonus-myoclonus syndrome (OMS), also known as thalamic myoclonus ataxia, is a syndrome consisting of thalamic myoclonus, diffuse or focal body myoclonus, and truncal titubation with or without ataxia and other cerebellar signs. In adults, OMS can be neoplastic or idiopathic [1], and underlying cancer is detected in 20-40% of cases [1, 2], with small cell lung cancer being the most commonly associated tumor [3]. The majority of paraneoplastic OMS (POMS) cases in adults are not associated with well-characterized antibodies [3]. Treating the underlying neoplasm in adults with POMS usually leads to the resolution of the neurologic symptoms [3]. Conversely, the occurrence of POMS due to non-small cell lung cancer (NSCLC) is extremely rare [4]. Herein, we report a case of paraneoplastic neurological syndrome (PNS) with refractory POMS from large cell neuroendocrine carcinoma (LCNEC), which is a rare case of NSCLC.

## Case presentation

A 60-year-old man with a 40 pack/year smoking history and an unremarkable medical history visited our hospital with a complaint of unsteadiness, dysarthria, and numbness on the right side of the body, which manifested the day before admission. The patient’s family was aware of his gait instability and progressive difficulty walking one week before admission. On physical examination, the patient demonstrated poor performance on the left side of the body in the finger-nose-finger test and knee-heel test. His joint gait was also poor, and a cerebellar lesion was initially suspected. Although magnetic resonance imaging (MRI) of the brain was normal, we suspected a cerebellar infarction, which was very subtle to be detected on imaging, as the cause of the cerebellar ataxia symptoms presenting on the left side. Therefore, we initiated antiplatelet and anticoagulation therapy. However, a few days after admission, the patient’s trunk ataxia, horizontal nystagmus, vertigo, and dysarthria worsened. Furthermore, the symptoms became bilateral. Owing to the exacerbation of the symptoms, multiple MRI scans the of brain with thin slices were performed, all of which showed normal results. Therefore, we considered cerebral infarction to be unlikely. However, irregular ocular rhythms and generalized involuntary movements appeared, indicative of OMS. We considered other possibilities, including autoimmune encephalopathies (e.g., Hashimoto’s encephalopathy), collagen diseases, vasculitis, infectious diseases (e.g., neurosyphilis and human immunodeficiency virus (HIV) encephalopathy), trace element deficiency and metabolic disorders, and PNS associated with malignant diseases. Most of these were ruled out after the various systemic examinations described below (Table 1).

**Table 1:** Laboratory findings WBC: white blood cells; RBC: red blood cells; Hb: hemoglobin; Ht: hematocrit; Plt: platelet; ESR: erythrocyte sedimentation rate; TP: total protein; Alb: albumin; T.Bil: total bilirubin; AST: aspartate aminotransferase; ALT: alanine aminotransferase; LDH: lactate dehydrogenase; BUN: blood urea nitrogen; Cre: creatinine; FBS: fasting blood glucose; CRP: C-reactive protein; VitB1: vitamin B1; TSH: thyroid-stimulating hormone; CEA: carcinoembryonic antigen; ProGRP: progastrin-releasing peptide; RPR: rapid plasma reagin; HTLV-1: human lymphotropic virus type 1; TPHA: treponema pallidum hemagglutination; HIV: human immunodeficiency virus; MPO-ANCA: myeloperoxidase-antineutrophil cytoplasmic antibody; PR3-ANCA: proteinase 3 antineutrophil cytoplasmic antibody; ADA: adenosine deaminase; HSV: herpes simplex virus; HHV3: human herpes virus 3.

Peripheral blood			Serology			Cerebrospinal fluid (CSF)		
WBC	6.9×10^3^	/μL	CRP	0.02	mg/dL	Cells	35	/mm^3^
Neutrophils	61.8	%	IgG	875	mg/dL	Mono	100	%
Eosinophils	1.3	%	IgA	125	mg/dL	Poly	0	%
Basophils	0.7	%	IgM	76	mg/dL	TP	32	mg/dL
Lymphocytes	30.7	%	IP	3.3	mg/dL	Glu	59	mg/dL
Monocytes	5.5	%	Mg	2.0	mg/dL	ADA	<2	IU/L
RBC	4.61×10^6^	/μL	Cu	109	μg/dL	Quantitative HSV-DNA	<1.0×10^2^	
Hb	15.9	g/dL	Zn	80	μg/dL	Quantitative HHV3-DNA	<1.0×10^2^	
Ht	45.0	%	VitB1	55.1	ng/mL	Oligoclonal band	(+)	
Plt	19.8×10^4^	/μL	TSH	1.040	mIU/L	Cytology	ClassⅠ	
ESR 1 hour	3	Mm	Free-T4	1.2	ng/dL			
			CEA	2.9	ng/mL	Anti-tumor antibody	Sero/CSF	
Biochemistry			CYFRA	2.18	ng/mL	Anti-Hu antibody	(-/-)	
TP	6.6	g/dL	ProGRP	27.0	pg/mL	Anti-Ri antibody	(-/-)	
Alb	4.3	g/dL	sIL-2R	513	U/mL	Anti-AMPH antibody	(-/-)	
T.Bil	0.8	mg/dL	T-spot	(-)		Anti-CV2/CRMP5 antibody	(-/-)	
AST	16	IU/L	HTLV-1	(-)		Anti-PNMA2 antibody	(-/-)	
ALT	18	IU/L	RPR	(-)		Anti-Yo antibody	(-/-)	
LDH	175	IU/L	TPHA	(-)		Anti-recoverin antibody	(-/-)	
BUN	10	mg/dL	HIV Ag-Ab	(-)		Anti-SOX1 antibody	(-/-)	
Cre	0.78	mg/dL	Anti-Tg antibody	(-)		Anti-titin antibody	(-/-)	
Na	139	mEq/L	Anti-TPO antibody	(-)		Anti-Zic4 antibody	(-/-)	
K	4.0	mEq/L	Anti-GAD antibody	(-)		Anti-GAD65 antibody	(-/-)	
FBS	114	mg/dL	Anti-AchR antibody	(-)		Anti-Tr (DNER) antibody	(-/-)	
HbA1c	5.3	%	Anti-GM1 antibody	(-)				
			Anti-GQ1b antibody	(-)				
			Anti-AQP4 antibody	(-)				
			Antinuclear antibody	<40				
			Anti-SS-A antibody	(-)				
			MPO-ANCA	(-)				
			PR3-ANCA	(-)				

**Figure 1:**
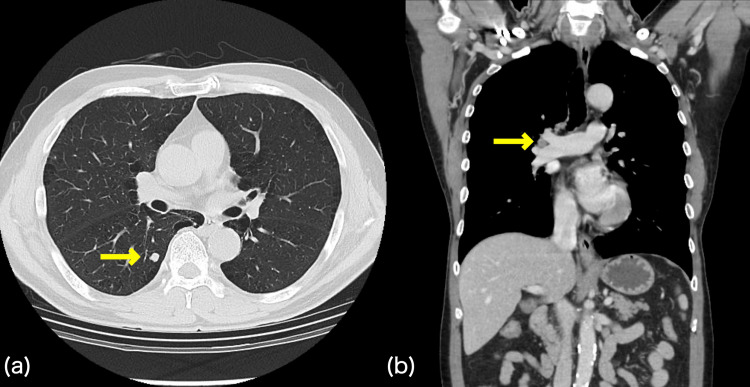
Chest computed tomography scan at the time of tumor detection (a) Axial view shows prominent emphysematous changes. A nodule sized 8 mm was found in S6 of the lower lobe of the right lung. There was no obvious calcification and no surrounding nodule or granular shadow. (b) Coronal view demonstrates small right hilum lymphadenopathy. (a, b) Based on the two findings, the patient was considered in clinical stage IIB (T1a, N1, M0).

**Figure 2:**
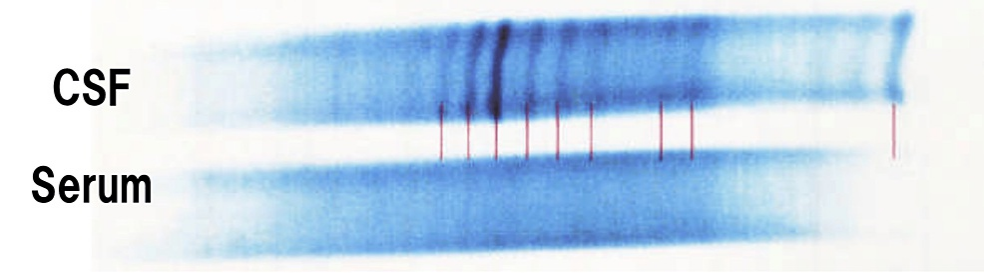
Oligoclonal bands detected using electrophoresis test Analysis of the electrophoretic pattern shows 30 oligoclonal bands, which are positive, suggesting some type of inflammatory neurological disease.

However, cervico-pelvic computed tomography (CT) revealed a small, 8-mm nodule in the right lower lobe (S6) and a mildly enlarged lymphadenopathy in the right hilum (Figure 1). Bone marrow examination showed significantly increased lymphocytes. Flow cytometry studies ruled out lymphoproliferative disease. After a pathological examination of spinal fluid specimens, the result was a mildly elevated cell count with lymphocyte predominance and a negative bacterial culture test, we concluded that the inflammatory changes were caused by encephalomyelitis. Electrophoresis of cerebrospinal fluid proteins revealed oligoclonal bands (Figure 2), suggesting the presence of some immunoglobulin protein. No PNS-related antibodies were identified in both blood and cerebrospinal fluid (CSF) (Table 1). Based on these findings, the final diagnosis was definitive PNS according to the guidelines [5].

**Figure 3:**
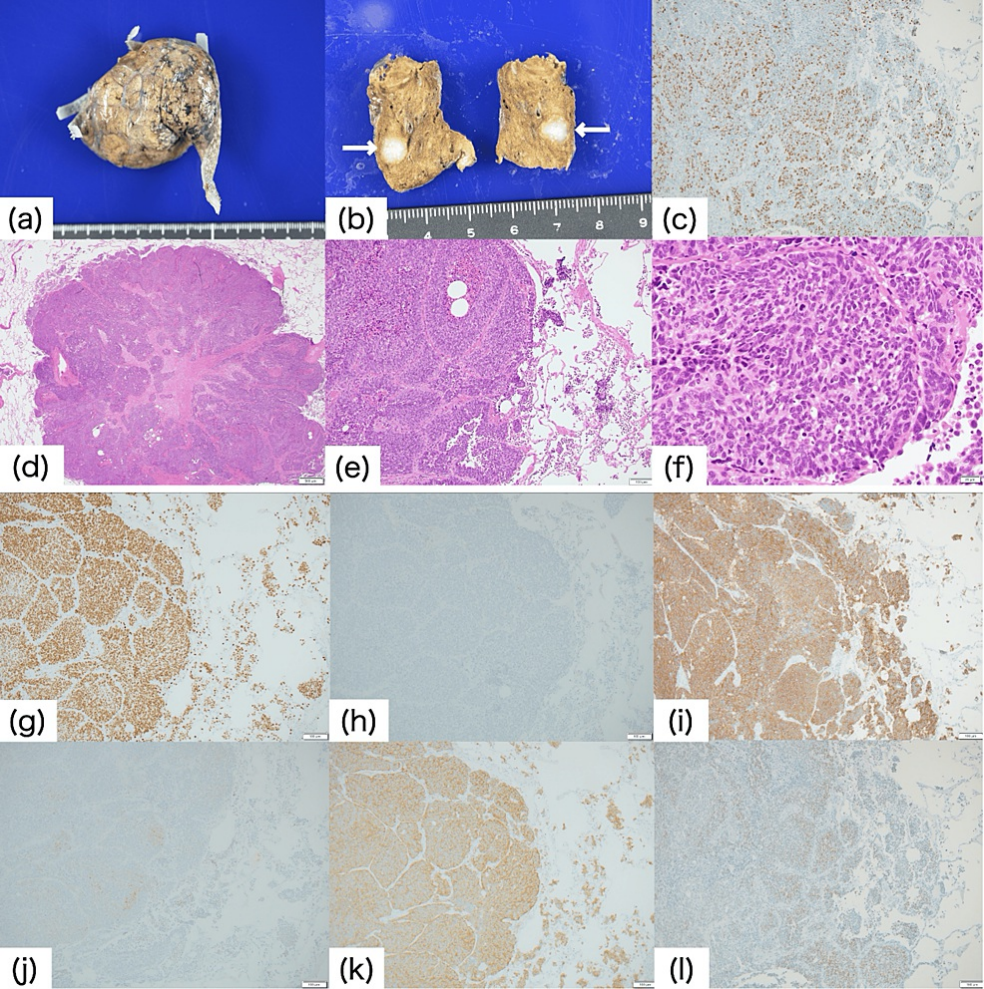
Histopathological findings of a partial lung resection specimen Grossly, there is a well-demarcated 7 × 6 × 6 mm white tumor. Histologically, it is composed of nests of atypical epithelial cells with increased nuclear/cytoplasmic ratio and enlarged hyperchromic nuclei. Necrosis is seen focally. Immunohistochemistry studies revealed a proliferation index by Ki67 of 60-70%. The tumor cells are positive for TTF-1 (g), CD56 (i), synaptophysin (k), and INSM-1 (l), focally positive for chromogranin A (j), and negative for p40 (h).

The patient was administered a three-day course of 1 g intravenous methylprednisolone and immunoglobulin therapy (0.4 g/kg/day); plasma exchange therapy was also administered; however, no improvement was noted. He underwent a right lower segmentectomy of the small nodule (S6) for diagnostic purposes. The results of a histopathological examination were compatible with high-grade LCNEC (Figure 3). After surgery, his symptoms worsened despite receiving anticonvulsant drugs such as clonazepam to alleviate his myoclonus symptoms. On the 5th postoperative day, he was intubated due to aspiration. The patient experienced relief from symptoms with deep sedation; however, his symptoms worsened upon waking. One week after intubation, extubation and withdrawal from the respirator were not feasible. Thus, a tracheostomy was performed. Two courses of chemotherapy with carboplatin and etoposide (70 mg/m2) were administered without improvement. Therefore, we proceeded to administer palliative care. At this time, his symptoms were relieved with 20 mg of clonazepam.

**Figure 4:**
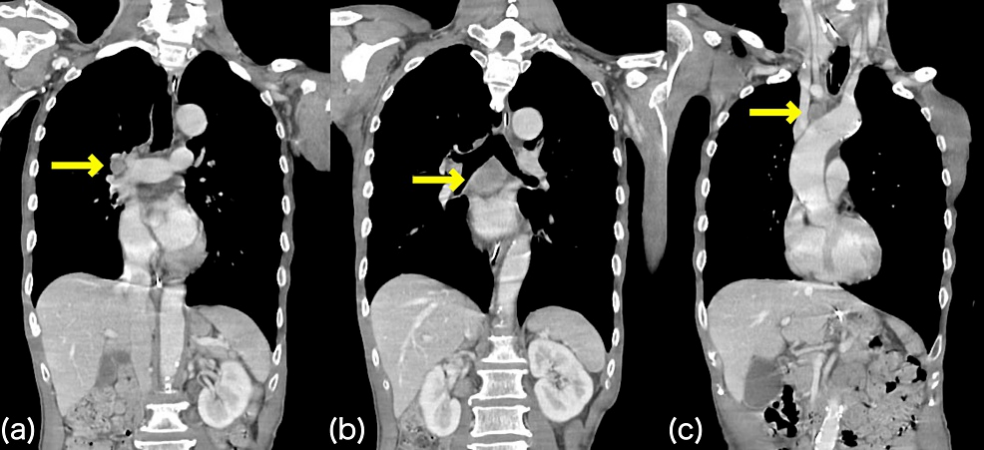
Recurrence eight months after onset Although chest computed tomography scans did not show any obvious mass, multiple lymphadenopathies were observed, and the diagnosis of recurrence was made. (a) Right hilar lymphadenopathy; (b) paratracheal lymphadenopathy; and (c) right upper paratracheal lymphadenopathy.

At five months after onset, only right hilum lymphadenopathy was detected, which was considered a stable disease. At six months after onset, the patient’s family reported his improvement and requested the removal of the respirator. He resumed ventilation on his own and eventually was able to wean himself off the mechanical ventilator and improved to the point of simple communication. In addition, the doses of anticonvulsant drugs were decreased. However, eight months after onset, the patient presented with poor vitality and poor communication, which led to repeated aspiration pneumonia. Since the pathological diagnosis was a high-grade LCNEC, we performed an imaging evaluation using CT. Although no obvious mass was detected, right upper paratracheal, right hilar, and subcarinal lymphadenopathy were demonstrated, all of which were considered multiple lymph node metastasis (Figure 4). No distant metastasis was found. He had a recurrence eight months after onset. And finally, he died the following month due to aspiration.

## Discussion

The aim of this report is to state that not only cerebrovascular disease but also PNS should be considered when treating patients with rapidly progressive cerebellar ataxia. Furthermore, one important and rare manifestation of PNS is the characteristic symptom of OMS; LCNEC is a rare cause of OMS and is refractory, and symptoms can be induced into remission before tumor recurrence.

POMS is a rare syndrome with an autoimmune pathogenesis that may be paraneoplastic or non-paraneoplastic [1,2]. Its etiology is not clearly defined; however, it is thought to be caused by the specific binding of various autoimmune antibodies to cerebellar nerves and Purkinje fibers [6], characterized by rare clinical manifestations of opsoclonus, localized or diffuse myoclonus of the trunk, and concurrent ataxic symptoms. POMS is one of the PNSs and one of the most important symptoms in the diagnostic criteria [5]. There are two approaches for the diagnosis of PNS, comprising both neurological syndrome and anti-neural antibody. In this case, OMS was the main symptom; however, anti-neural antibodies, such as anti-Hu, anti-Yo, anti-CV/CRMP5, anti-Ri antibody, anti-Ma2, and anti-amphiphysin antibodies were not detected. However, the presence of multiple classic neurological syndromes, such as OMS, and the presence of a tumor suggested the diagnosis of definite PNS. Adults over 50 years of age and those with associated encephalopathy should be rigorously evaluated for occult malignancy.

The tumor that is most frequently associated with POMS in adults is small cell lung carcinoma (SCLC) [2]. LCNEC is a rare pulmonary tumor with features of NSCLC. Because LCNEC is rare, there are no large trials to define the optimal treatment approach for localized or advanced disease [7], and most studies have shown that stage-matched survival rates for LCNEC are worse than for other NSCLC and other large-cell cancers, and have a similar poor prognosis as survival rates for SCLC [8]. Although there have been no reports of OMS in LCNEC, this case indicates that LCNEC may be a candidate for the cause of OMS.

Some adults with POMS experience resolution of neurologic symptoms after being treated for the underlying neoplasm. Compared with children, adults are less likely to respond to immunotherapy [9]. In some cases, patients respond to treatment with high doses of clonazepam after failing to respond to immunotherapy [10]. In this case, the patient’s management was difficult; however, we successfully controlled his symptoms by treating the underlying neoplasm and administering immunotherapy and anticonvulsants. In this case, the patient had a recurrence seven months after his first diagnosis. We had concerns regarding the potential worsening of his symptoms; unfortunately, they did worsen subacutely.

In summary, we have experienced a case of LCNEC diagnosed with OMS. When treating patients with rapidly progressive cerebellar ataxia, PNS as well as cerebrovascular disease should be considered, and OMS should not be forgotten as a manifestation of PNS. This case also showed that OMS can be one of the symptoms of LCNEC, that it is refractory, and that it can still induce remission of symptoms until the tumor recurrence.

## Conclusions

We considered PNS in a patient with neurological symptoms of POMS and diagnosed LCNEC after a systemic search. LCNEC has been reported as a rare cause of OMS. Although we initially suspected cerebellar infarction based on the neurological symptoms, it is important for early diagnosis to consider the possibility of PNS when rapidly progressive cerebellar symptoms are observed. The clinical course of this case suggests that PNS due to LCNEC may be refractory. In this case, a combination of all possible treatments, including surgical treatment, chemotherapy, and immunological treatment, resulted in a delayed clinical response. We encountered a case of an uncommon disease with an uncommon presentation, and the fact that we were able to induce a state of remission before relapse is a crucial finding.
